# Role of miR-101a in targeting Cox-2 to attenuate chondrocyte hypertrophic differentiation and osteoarthritis progression

**DOI:** 10.1016/j.gendis.2025.101839

**Published:** 2025-09-01

**Authors:** Rui Mi, Jinnan Chen, Tianxiang Zhu, Huiqin Bian, Rong Wei, Rushuang Deng, Tiaotiao Han, Qian Wang, Yaojuan Lu, Longwei Qiao, Yuting Liang, Qiping Zheng

**Affiliations:** a Department of Hematology and Hematological Laboratory Science, Jiangsu Key Laboratory of Medical Science and Laboratory Medicine, School of Medicine, Jiangsu University, Zhenjiang, Jiangsu 212013, China; b Department of Clinical Laboratory, Wuxi Ninth People's Hospital Affiliated to Soochow University, Wuxi, Jiangsu 214000, China; c Department of Human Anatomy, School of Medicine, Jiangsu University, Zhenjiang, Jiangsu 212013, China; d Shenzhen International Institute for Biomedical Research, Shenzhen, Guangdong 518110, China; e Shenzhen Walgenron Bio-Pharm Co., Ltd., Shenzhen, Guangdong 518118, China; f The Affiliated Suzhou Hospital of Nanjing Medical University, Nanjing, Jiangsu 215008, China; g The Molecular Oncology Laboratory, Department of Orthopedic Surgery and Rehabilitation Medicine, The University of Chicago Medical Center, Chicago, IL 60637, USA; h Center for Clinical Laboratory, The First Affiliated Hospital of Soochow University, Suzhou, Jiangsu 215000, China

**Keywords:** Chondrocyte differentiation, *Col10a1*, *Cox-2*, miR-101a, Osteoarthritis

## Abstract

MicroRNAs (miRNAs) are small non-coding RNAs that regulate gene expression post-transcriptionally, often playing critical roles in various biological processes. Recent studies have highlighted the involvement of miRNAs in chondrogenesis by targeting key marker genes. Among these, miR-101a has been identified as a significant regulator, previously reported to target cyclooxygenase-2 (Cox-2, ptgs2) in various contexts. Here, we investigate the role of miR-101a in chondrocyte hypertrophy and osteoarthritis (OA) progression, focusing on its regulation of Col10a1 expression. Using multiple web-based tools (TargetScan, PicTar, miRDB, and miRCODE), we identified miR-101a as a potential regulator of Col10a1. Our *in vitro* experiments demonstrated that miR-101a was down-regulated during chondrocyte hypertrophy in MCT and ATDC5 cells, while Col10a1 and Cox-2 expression levels were up-regulated. Overexpression of miR-101a via mimics resulted in a significant decrease in Col10a1 and Cox-2 at both mRNA and protein levels, whereas inhibition of miR-101a led to their up-regulation. Additionally, MMP-13 protein levels were reduced upon miR-101a overexpression, with no significant changes in Sox9 and Runx2 expression. Luciferase reporter assays confirmed that Cox-2 was a direct target of miR-101a, suggesting that miR-101a indirectly regulates Col10a1 expression via Cox-2. *In vivo*, intra-articular injection of miR-101a mimics in a medial meniscus-induced OA mouse model resulted in decreased Col10a1 expression and reduced articular damage, supporting the protective role of miR-101a in OA progression. Our findings highlight miR-101a as a negative regulator of chondrocyte hypertrophy through Cox-2, and could be a potential target for further exploration in OA therapy.

## Introduction

Osteoarthritis (OA) is a prevalent chronic degenerative joint disease, primarily affecting middle-aged and elderly individuals. It is characterized by progressive cartilage degradation, subchondral bone remodeling, and synovial inflammation.^1^^,^^2^ A hallmark of OA pathology is the aberrant hypertrophic differentiation of chondrocytes, a process typically associated with the terminal stage of endochondral ossification.^3^^,^^4^ This phenotypic shift contributes to extracellular matrix breakdown and cartilage degeneration. Despite its pivotal role in OA progression, chondrocyte hypertrophy remains an under-targeted process in current therapeutic strategies, which predominantly focus on symptom management.^5^

Among the genes implicated in hypertrophic differentiation, collagen type X alpha 1 (*Col10a1*) is a well-established marker.^6^^,^^7^ Abnormal expression of *Col10a1* is associated not only with OA but also with skeletal disorders such as Schmid-type metaphyseal chondrodysplasia, characterized by growth plate abnormalities and skeletal deformities.^8^ Mechanistic insights into the regulation of *Col10a1* are critical not only for understanding OA pathogenesis but also for advancing regenerative approaches using *in vitro* cartilage models and organoids.^9^^,^^10^ Cyclooxygenase-2 (*Cox-2*, *Ptgs2*) is another key player in skeletal biology and OA pathology. As an inducible enzyme involved in prostaglandin synthesis, *Cox-2* mediates inflammation, fracture healing, and endochondral ossification.^11^^,^^12^ Elevated *Cox-2* levels in OA cartilage promote extracellular matrix degradation and inflammation via prostaglandin E2 (PGE2) signaling,^13^^,^^14^ while *Cox-2* knockout models exhibit impaired skeletal repair and reduced expression of transcription factors such as Runt-related transcription factor 2 (Runx2) and specificity protein 7 (Sp7).^15^

MicroRNAs (miRNAs), a class of ∼21-nucleotide non-coding RNAs, regulate gene expression at the post-transcriptional level and are essential for skeletal development and cartilage homeostasis.^16^^,^^17^ Dicer-knockout mice, which are deficient in miRNA biogenesis, show severe skeletal defects, underscoring the crucial role of miRNAs in cartilage formation.^18^^,^^19^ Several miRNAs have been identified as regulators of chondrocyte hypertrophy and OA-related pathways. For instance, miR-26a and miR-199a inhibit hypertrophic and inflammatory responses by targeting *Col10a1* and *Cox-2*, respectively.^20^^,^^21^ Others, such as miR-140, miR-146a, miR-26b-5p, and miR-27b, modulate key OA-related genes, including matrix metallopeptidase 13 (*MMP-13*), *COL2A1*, *RUNX2*, interleukin-1beta (*IL-1β*), ADAM metallopeptidase with thrombospondin type 1 motif 5 (*ADAMTS-5*), and *MMP-1*.^22^, ^23^, ^24^, ^25^, ^26^ *miR-101* has emerged as a multifunctional regulator in cartilage and bone biology. Previous studies have shown its involvement in cartilage degradation via the dual specificity phosphatase 1 (DUSP1) pathway in rheumatoid arthritis, modulation of senescence and extracellular matrix breakdown through the LINC00623/miR-101/HRAS axis, and inhibition of SRY-box transcription factor 9 (*SOX9*), affecting chondrocyte survival and differentiation.^27^, ^28^, ^29^, ^30^ Moreover, *miR-101* influences cartilage integrity via epigenetic regulation of integrin-α1 and can prevent degradation when suppressed in OA models.^31^^,^^32^ However, its specific role in regulating chondrocyte hypertrophic differentiation has not been fully elucidated.

In our previous work, we demonstrated that *Cox-2* promoted *Col10a1* expression and contributed to chondrocyte hypertrophy.^33^ Given their similar expression profiles in hypertrophic chondrocytes, we hypothesized that both *Cox-2* and *Col10a1* may be co-regulated by a common miRNA. Through bioinformatics analysis and experimental validation, we identified *miR-101a* as a direct regulator of *Cox-2*, indirectly of *Col10a1*. *In vitro*, *miR-101a* levels decreased during hypertrophic differentiation of MCT and ATDC5 cells, while overexpression of *miR-101a* reduced *Cox-2*, *Col10a1*, and *MMP-13* expression, without significantly affecting *SOX9* and *RUNX2*. Luciferase reporter assays confirmed *Cox-2* as a direct target of *miR-101a*. Importantly, intra-articular injection of *miR-101a* mimics in a destabilization of the medial meniscus (DMM)-induced OA mouse model led to reduced *Col10a1* expression and attenuation of articular cartilage damage. These findings establish *miR-101a* as a novel modulator of chondrocyte hypertrophy via direct targeting of *Cox-2* and suggest its therapeutic potential in OA intervention.

## Materials and methods

### Cell lines and cell culture

The mouse cartilage-derived MCT cell line was kindly provided by Dr. B. de Crombrugghe's laboratory at MD Anderson Cancer Center (Houston, Texas, USA), and the mouse cartilage-like ATDC5 was originally from the department of orthopedic surgery at New York University Medical Center. The MCT cells were maintained in Dulbecco's Modified Eagle Medium (DMEM, BI, Israel), supplemented with 8% fetal bovine serum (BI, Israel). The temperature-sensitive MCT cells were cultured at 32 °C (proliferative) in a humidified incubator containing 5% CO_2_ and acquired hypertrophy-like performance when the temperature was up to 37 °C. Mouse cartilage-like ATDC5 cells were cultured in DMEM/F12 (HAM) (1:1) with 5% fetal bovine serum (proliferative) and were treated with 1 × insulin-transferrin-sodium selenite (ITS, Sigma I3146, Germany) to induce hypertrophy.

### Overexpression and down-regulation of miRNA

The 5′ FAM chemically modified miR-101a mimics, miR-101a mimics negative control (mimic NC), miR-101a inhibitor, and miR-101a inhibitor negative control (inhibitor NC) were synthesized by GenePharma (Shanghai, China). MCT cells were transfected with 50 nM of either miR-101a mimics, miR-101a inhibitors, or their respective negative controls (mimic NC and inhibitor NC), in 24-well plates using 2 μL Lipofectamine™ 2000 (Invitrogen, USA) according to the manufacturer's protocol. The transfected cells were subjected to 37 °C after 6 h and then cultured in the complete medium for 48 h. To optimize transfection conditions, additional experiments were performed using 40 nM and 80 nM of miR-101a mimics and 80 nM and 120 nM of miR-101a inhibitors. After 6 h of transfection at 37 °C, cells were incubated in complete medium and harvested at 24, 48, or 72 h post-transfection for quantitative reverse transcription PCR or western blotting analysis. Lentiviral vectors containing miR-101a mimics, miR-101a inhibitors, and negative controls were all commercially synthesized by GenePharma (Shanghai, China). ATDC5 cells were transduced with L/miR-101a mimic, L/miR-101a inhibitor, and L/C at a multiplicity of infection (MOI) of 0.6 × 10^8^ TU/mL for 24 h. Cells were maintained in DMEM/F12 medium supplemented with 5% fetal bovine serum and 5 μg/mL streptomycin (Sigma, USA). Colonies were picked up from survival colonies after puromycin selection for 2 weeks. It was confirmed to have integrated with miR-101a mimic and miR-101a inhibitor expression plasmid, and used for subsequent experiments. miR-101a mimic and miR-101a inhibitor stable expression ATDC5 cell line was serially cultured with an optimized concentration of ITS. For time-course validation, stable ATDC5 lines were collected on day 7 and day 21 of ITS-induced differentiation, and subjected to analysis of miR-101a expression and downstream gene regulation.

### RNA isolation and quantitative PCR

The total RNA of proliferative and hypertrophic MCT and ATDC5 cells was isolated with TRIzol® reagent (Invitrogen, Thermo Fisher Scientific, Inc., USA) following the manufacturer's instructions. After spectrophotometric quantification, 1 μg of total RNA in a final volume of 20 μL was used for reverse transcription with a PrimeScript™ RT reagent Kit with gDNA Eraser (Perfect Real Time) (TaKaRa, Japan) according to the manufacturer's protocols. Hairpin-it™ miRNAs qPCR Quantitation Kit (GenePharma, Shanghai, China) was used to examine the expression of miR-101a. Reverse transcription quantitative real-time PCR was performed with ultra-SYBR Premix (CWBIO, China) by the Applied Biosystems QuantStudio 5 PCR instrument (ThermoFisher Scientific, USA) according to the thermocycling conditions. *β-actin* was used as an internal control of mRNA, and U6 was used for miRNA. The 2^–ΔΔCt^ method was used to calculate the fold change of the target genes. Further details of primer sequences used in our study are shown in Table 1.

**Table 1:** Primers for PCR and miRNA fragment.

List of oligonucleotide sequences		
Gene	Sequence (5′–3′)	
*Gapdh*	ACCCAGAAGACTGTGGATGG	Forward
*Gapdh*	CACATTGGGGGTAGGAACAC	Reverse
*Cox-2*	TGCAGAATTGAAAGCCCTCT	Forward
*Cox-2*	CCCCAAAGATAGCATCTGGA	Reverse
*Col10a1*	TCTGTGAGCTCCATGATTGC	Forward
*Col10a1*	GCAGCATTACGACCCAAGATC	Reverse
*Mmp13*	CTTCTTCTTGTTGAGCTGGACTC	Forward
*Mmp13*	CTGTGGAGGTCACTGTAGACT	Reverse
*Runx2*	ACCCAGCCACCTTTACCTAC	Forward
*Runx2*	TATGGAGTGCTGCTGGTCTG	Reverse
*Sox9*	TTCATGAAGATGACCGACGA	Forward
*Sox9*	ATGCACACGGGGAACTTATC	Reverse
U6	CTCGCTTCGGCAGCACA	Forward
U6	AACGCTTCACGAATTTGCGT	Reverse
miR-101a-3p	CATCGCACGTACAGTACTGTGATA	Forward
miR-101a-3p	GTGCAGGGTCCGAGGT	Reverse
*miRNA agomir fragments*		
agomir Negative control (NC)	UUUGUACUACACAAAAGUACUG	Sense
agomir Negative control (NC)	CAGUACUUUUGUGUAGUACAAA	Anti-sense
Mmu-miR-101a-3p agomir	UACAGUACUGUGAUAACUGAA	Sense
Mmu-miR-101a-3p agomir	UUCAGUUAUCACAGUACUGUA	Anti-sense

### Western blotting

Total cellular protein was extracted with radioimmunoprecipitation assay buffer (Beyotime, China) supplemented with 1 × phenylmethylsulfonyl fluoride (PMSF, Beyotime, China), and the supernatant was collected and quantified by a spectrophotometer. An equal amount of protein extracts (100 μL) was separated by 10% SDS-PAGE gel at 100 V for 1.5–2 h and then transferred to the 0.22 μm PVDF membrane (Millipore, Billerica, Massachusetts, USA) at 350 mA for 90 min. The membranes were incubated with specific primary antibodies at 4 °C overnight after blocking with 5% fat-free milk at room temperature for 2 h. The next day, after washing the membrane three times with 1 × Tris-buffered saline with Tween 20 for 15 min, the membrane was incubated with the corroding horseradish peroxidase-labelled secondary antibodies at room temperature for 1 h. Finally, the target bands were visualized by chemiluminescence using a GE Amersham Imager 600. The primary antibodies included COL10A1 (ab182563, Abcam, Massachusetts, USA), COX-2 (D223097, Biotechnology, Shanghai, China), MMP13 (ab51072, Abcam, Cambridge, Massachusetts, USA), RUNX2 (#12556, CST, USA), and SOX9 (#82630, CST, USA), which were prepared with primary antibody dilution (Yeasen Biotechnology, China). β-ACTIN was used as an internal control.

### EdU proliferation assay

Proliferative MCT and ATDC5 cells were transfected with miRNA mimics, inhibitors, and controls in 96-well plates. The cells were collected after 48 h and then were detected according to the manufacturer's instructions (Cell Light EdU DNA imaging Kit, Guangzhou, RiboBio, China). Briefly, cells were cultured with 10 μM EdU for 2 h before fixation, permeabilization, and staining.

### Alcian blue, alizarin red, and alkaline phosphatase staining

ATDC5 cells from the miR-101a stable line and controls undergoing differentiation were measured by Alcian blue staining to assess the deposition of cartilage matrix proteoglycans. The cells used for Alcian blue staining were fixed with methanol at −20 °C for 2 min. Then, they were stained with 0.1% Alcian blue (Biotechnology, Shanghai, China) overnight. The induced ATDC5 cells were stained with alizarin red to detect the Calcium deposition. Cells were fixed with 95% ethanol for 10 min and then stained with 1% alizarin red. The induced ATDC5 cells were also collected to analyze calcium deposition. Cells were stained according to the manufacturer's instructions for the CAKP kit (Jiancheng, Nanjing, China).

### Dual-luciferase reporter gene assay

293T cells were co-transfected with 0.5 μg of either the pirGLO-*Col10a1* 3′ UTR-WT, pirGLO-*Col10a1* 3′ UTR-MUT, and 50 nM of either miRNA mimics negative control or miR-101a mimics. *Cox-2* plasmids were treated in the same way. After the transfection for 24 h, the obtained cells were used for the luciferase assay and subsequent luminescence calculations. Then, the Firefly and Renilla luciferase were measured with a Dual-Glo luciferase reporter assay system (Promega, E1960, Wisconsin, USA).

### Induction of mouse knee OA model and intra-articular injection

Animal experiments were performed under the approval of the Animal Experimentation of Jiangsu University and according to the guidelines of the National Institutes of Health Guide for the Care and Use of Laboratory Animals. C57BL/6 male mice at 4 weeks old were purchased from Changzhou Cavens Laboratory Animal Co., Ltd., Jiangsu, China.

The mouse knee OA model was induced by DMM surgery according to the previously described procedure.^34^^,^^35^^,^^36^ Briefly, the mouse was anesthetized via intraperitoneal injection of sodium pentobarbital (Sigma–Aldrich, St. Louis, Missouri, USA) at a dose of 50 mg/kg, and the surgical procedure was conducted for DMM in their left knee joints. Postoperatively, chlortetracycline ointment was coated on the incision to prevent infection. The timeline for these studies is depicted in Figure 7A. Two weeks after surgery, mice in the DMM group were randomly divided into groups treated with intra-articular injections of miR101a agomir and negative control. The miR101a agomir and negative control are synthesized and shipped as powders, which should be diluted with 15 μL (5 nmol miRNA agomir) saline and completely solubilized in a 37 °C water bath before use.^37^^,^^38^ Two intra-articular injections were performed at 2 and 4 weeks postoperatively, the mice were sacrificed 8 weeks after the completion of the injections, and the left knee joint samples were collected and embedded in paraffin for histological analysis.

**Figure 1:**
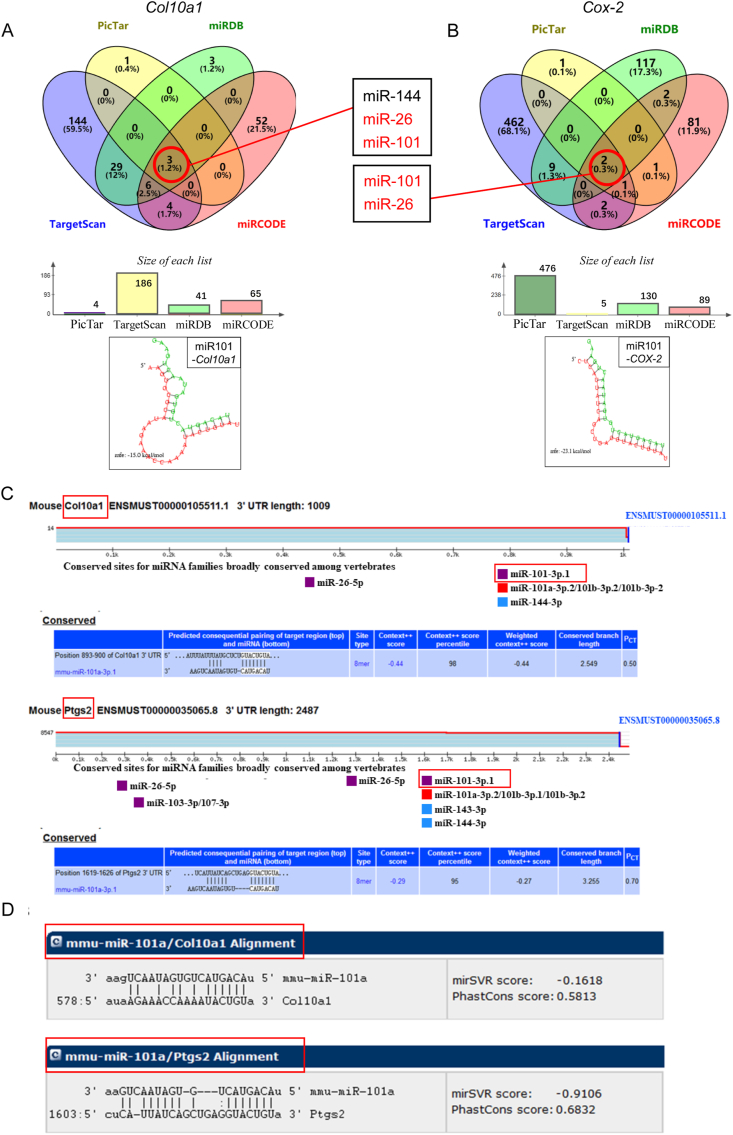
Bioinformatics prediction of interactions between microRNAs and target genes. **(A, B)** The Venn diagram displaying miR-101a-3p computationally predicted to target *Col10a1* and *Cox-*2 by four different prediction algorithms: TargetScan, PicTar, miRDB, and miRCODE. The structure of the predicted duplex by PicTar was shown. **(C)** TargetScan bioinformatics prediction result of *Col10al* and *Ptgs2* (*Cox-2*) was performed. **(D)** miRanda Bioinformatics prediction result of *Col10a1* and *Ptgs2* (*Cox-2*).

**Figure 2:**
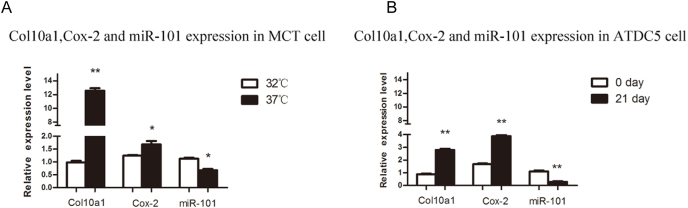
*Col10a1*, *Cox-2*, and miR-101a expression in cell lines of chondrocyte hypertrophy. **(A)** The levels of *Col10a1*, *Cox-2,* and miR-101a were analyzed in proliferative (32 °C) and hypertrophic (37 °C) MCT cells. The expression levels of *Col10a1* and *Cox-2* significantly increased compared with proliferative MCT cells, whereas miR-101a exhibited weak expression during chondrocyte hypertrophy. **(B)** The expression levels of *Col10a1*, *Cox-2*, and miR-101 were shown in the ATDC5 cell line. Both *Col10a1* and *Cox-2* levels were up-regulated during 21-day 1% ITS induction. However, miR-101 had lower expression in chondrocyte hypertrophy. ∗*P* < 0.05 and ∗∗*P* < 0.01.

**Figure 3:**
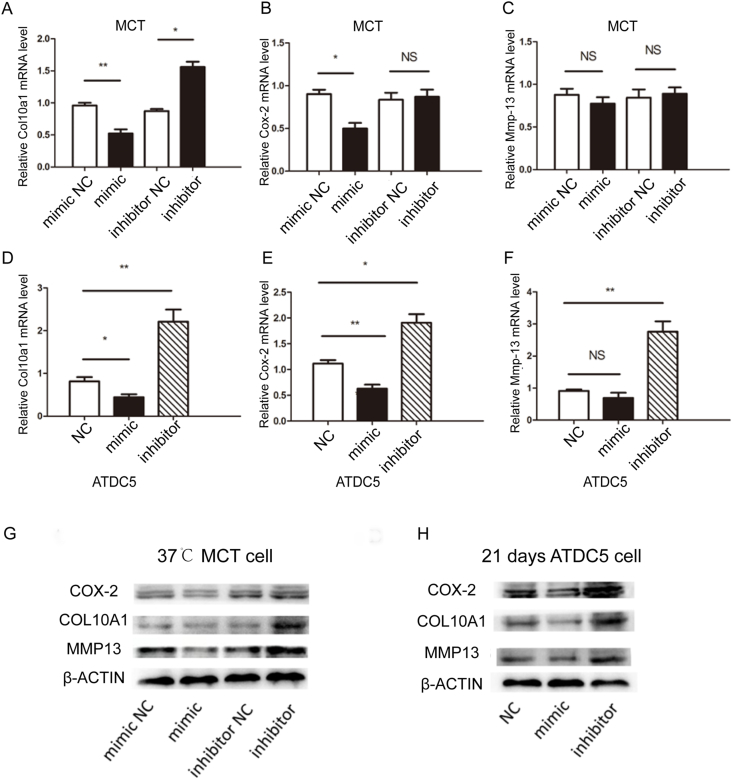
miR-101a inhibits hypertrophic differentiation of chondrocytes *in vitro*. **(A**–**C)** Relative mRNA levels of *Col10a1*, *Cox-2*, and *Mmp13* after transfected with miR101a mimic in ATDC5 cells. **(D**–**F)** Relative mRNA levels of *Col10a1*, *Cox-2*, and *Mmp13* after transfected with miR101a mimic in MCT cells. **(G)** The protein levels of COL10A1, COX-2, and MMP13 after miR-101a interference in MCT cells were investigated by western blotting. **(H)** The protein levels of COL10A1, COX-2, and MMP13 after miR-101a interference in hypertrophy-induced ATDC5 cells were detected by western blotting.

**Figure 4:**
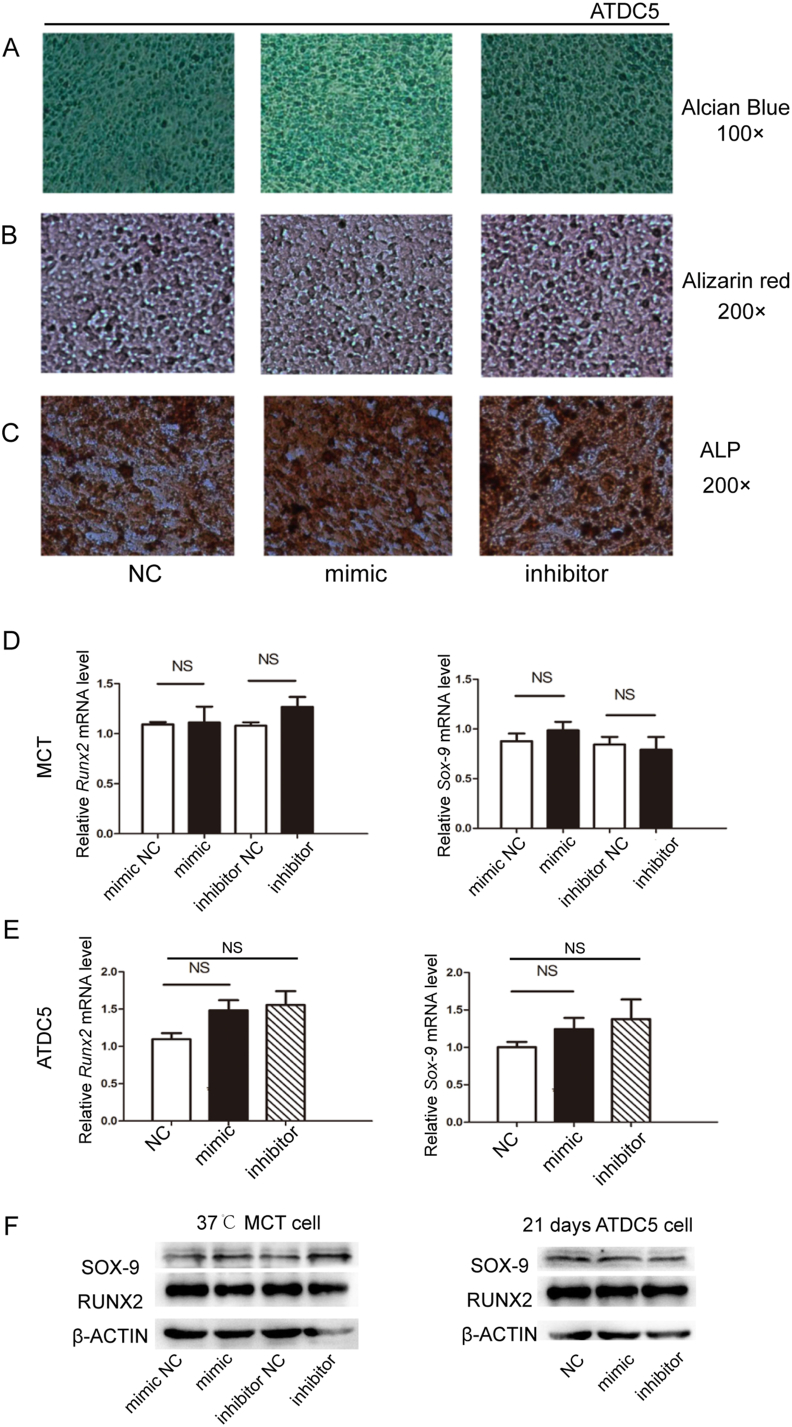
The effects of miR-101a on chondrogenic differentiation in ATDC5 cells. **(A)** Representative pictures of Alcian blue staining of ATDC5 cells after transfected with miR101a mimic and inhibitor. Magnification, 100×. **(B, C)** Representative pictures of Alizarin red staining and alkaline phosphatase (ALP) staining of ATDC5 cells after being transfected with miR-101a mimic and inhibitor. Magnification, 200×. **(D, E)** Reverse transcription quantitative real-time PCR analysis was utilized to evaluate the mRNA expression of *Runx2* and Sox-9 in MCT and ATDC5 cells following miR-101a interference. **(F)** The protein levels of RUNX2 and SOX-9 were assessed by western blotting.

**Figure 5:**
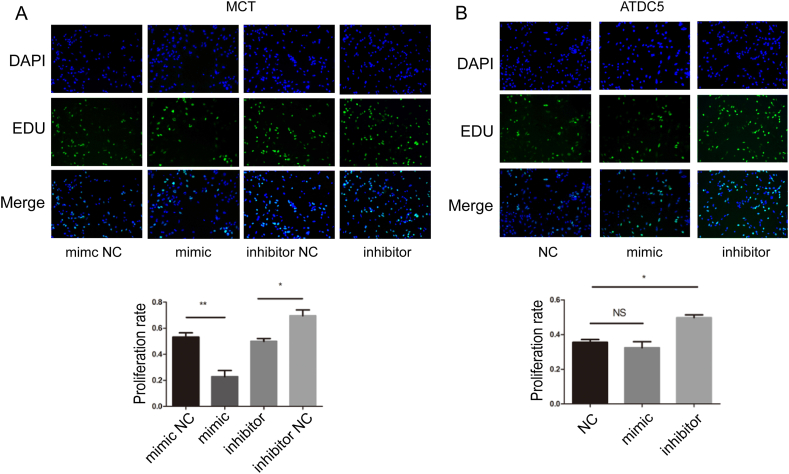
Overexpression of miR-101a inhibits the proliferation ability of chondrocytes. **(A)** The representative images of fluorescence and the percentage of EDU-positive cells following the transfection of MCT with miR101a mimic and inhibitor. **(B)** The representative fluorescent images of ATDC5 cells transfected with miR-101a mimic and inhibitor. The nucleus was labeled with Hoechst 33342 (blue fluorescence), while the proliferative cell was labeled with EdU (green fluorescence).

**Figure 6:**
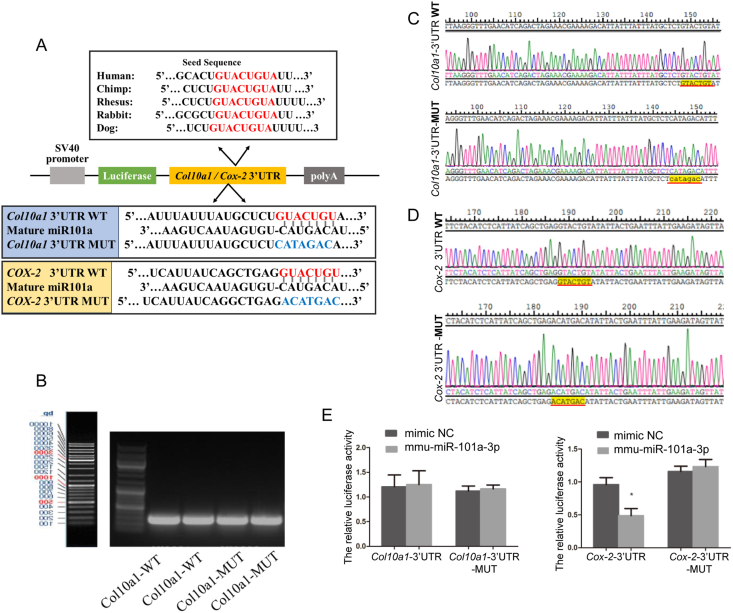
Validation of the targeting effect of miR-101a on *Col10a1* and *Cox-2.***(A)** Schematic diagram of the WT and MUT luciferase reporter plasmid constructs. The mutation sequences were generated in the *Col10a1* and *Cox-2* 3′ UTR sequence in the complementary site for the seed region of miR-101a, as indicated. The potential binding sequences are indicated in red, while the sites of mutation are shown in blue. **(B)** The amplification products of *Col10a1*-miR-101a-WT and *Col10a1*-miR-101a-MUT were obtained by conventional PCR, and the results of agarose gel electrophoresis showed that the fragment sizes were around 230 bp. **(C)** Sanger sequencing was utilized to identify the precision of the mutation site on the *Col10a1* reporter plasmid. **(D)** Sanger sequencing was utilized to identify the precision of the mutation site on the *Cox-2* reporter plasmid. **(E)** Luciferase activity of the *Col10a1* and *Cox-2* 3′ UTR reporter was analyzed in 293T cells. ∗*P* < 0.05.

**Figure 7:**
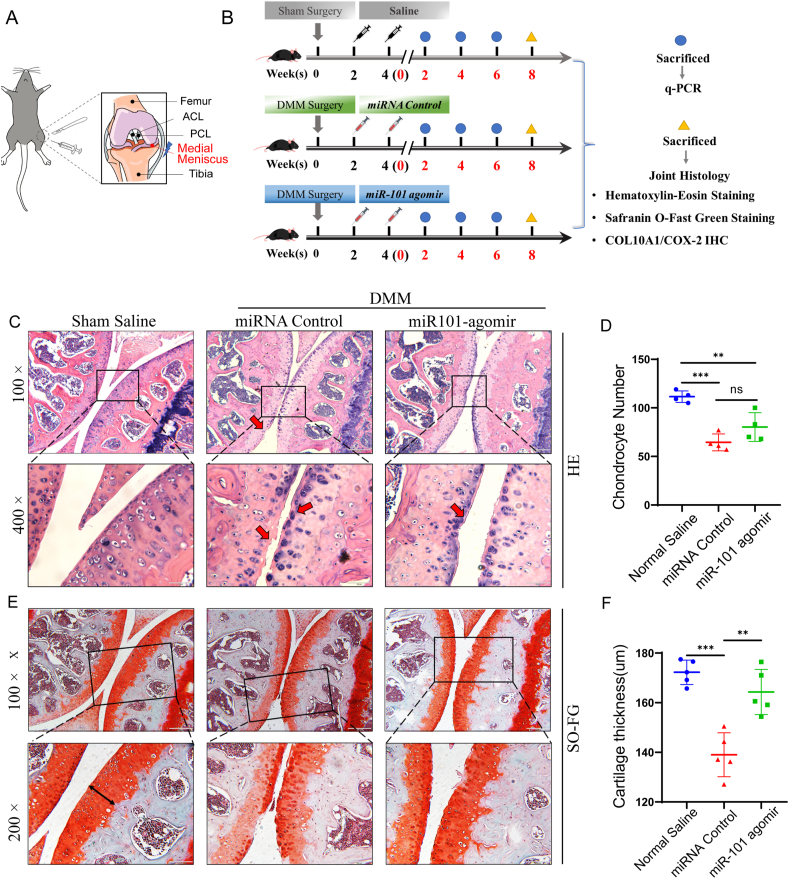
Intra-articular injection of miR-101a attenuated OA progression. **(A)** The diagram illustrates the destabilization of the medial meniscus (DMM) surgical process. Six-week-old male C57BL/6J mice underwent DMM surgery on the medial meniscus of the left hind limb. The medial meniscus tibial ligament, with a portion of the medial meniscus, was dissected by microsurgery. PCL, posterior cruciate ligament; ACL, anterior cruciate ligament. DMM surgery is indicated by blue lightning. **(B)** The graphic demonstrates the timeline of the postoperative injection procedure of the three groups of mice. Thirty-three model mice were randomly allocated into three groups (*n* = 11 in each). After three weeks of surgery, mice were treated with equal amounts (15 μL) of normal saline (sham/saline), miRNA control (5 nmol), and miRNA-101 agomir (5 nmol) via intra-articular injection administered through a medial parapatellar approach (the mice in the sham group underwent sham surgery on the right knee). Mice were sacrificed at 2, 4, 6, and 8 weeks after injection for corresponding molecular biological and histological analyses. **(C)** The hematoxylin and eosin staining of the knee joint coronal sections. Magnification = 100× and scale bar = 200 μm; magnification = 400× and scale bar = 50 μm. **(E)** The safranin O/fast green staining of the knee joint coronal sections. Magnification = 100× and scale bar = 200 μm; magnification = 200× and scale bar = 100 μm. **(D)** The statistical count of the chondrocyte number was determined by hematoxylin and eosin staining (thick red arrow: from the cartilage surface to the deep zone). **(F)** The knee joint cartilage thickness was determined by safranin O/fast green staining (black double arrow: from the cartilage surface to the deep zone). ∗*P* < 0.05, ∗∗*P* < 0.01, and ∗∗∗*P* < 0.001.

### Histological staining and assessment

The paraffin blocks were cut into 5 μm serial frontal plane sections for the following staining with either hematoxylin, fast green/safranin O, or hematoxylin/eosin. Five different fields of view were used to quantify chondrocytes. The width of cartilage in safranin O/fast green staining slides was measured to operationalize knee joint damage. All the evaluations were independently performed by two researchers.

### Statistical analysis

All detections were based on at least three individual experiments. Differences between the groups were determined using the Student's *t*-test. A one-way analysis of variance (ANOVA) test was used to evaluate whether a gross statistically significant change existed. Data were represented as mean ± standard error of the mean, and *P* values < 0.05 or < 0.01 were considered statistically significant. All the data were analyzed using GraphPad Prism software version 8.0.

## Results

### Bioinformatics prediction of a single miRNA that targets Col10a1 and Cox-2

To find a single miRNA that regulates the expression of *Col10a1* and *Cox-2*, four kinds of online bioinformatics approaches were used to screen the miRNAs that could interact with the 3′ UTR of both mRNAs separately (TargetScan, PicTar, miRDB, and miRCODE). Through screening the 3′ UTR region of *Col10a1* and *Cox-2* from four miRNA target databases, we identified two overlapping candidate miRNAs, miR101 and miR26, and the structure of the predicted duplex by PicTar was shown (Fig. 1A and B). miR-26a was proven to regulate *Col10a1* gene expression in previous studies. miR-26a could directly bind to and inhibit the expression of *Col10a1* in primary epiphyseal chondrocytes and manipulate chondrocyte hypertrophy and the cartilage extracellular matrix composition.^20^ We further confirmed the binding site of miR101 with the 3′ UTR of *Col10a1* and *Cox-*2 by TargetScan and miRanda, and the results were consistent with the predicted site of Pictar, and there existed complementary sites in the seed region of miR-101a with *Cox-2* and *Col10a1* (Fig. 1C and D).

### Expression levels of miR-101a, Col10a1, and Cox-2 in proliferative and hypertrophic chondrocyte models

The MCT cells were mouse chondrocytes that were immortalized with a temperature-sensitive simian virus 40 large tumor antigen.^39^ The MCT cells proliferated continuously when cultured at 32 °C, while the culture temperature was raised to 37 °C, the cell proliferation rate decreased, and the cells underwent a hypertrophic differentiation phase with specific expression of the collagen type X gene. In response to changes in culture temperature, MCT cells can mimic the differentiation process of chondrocytes *in vitro* and are a classic cell model for *in vitro* studies of chondrocyte differentiation and maturation. The expression changes of miR-101a, *Col10a1*, and *Cox-2* both in the proliferative (32 °C) and hypertrophic (37 °C) stages were detected by quantitative PCR. The results showed that the *Col10a1* mRNA level was higher in hypertrophic differentiation compared with proliferative MCT cells. Furthermore, *Cox-*2 mRNA is two-fold higher in hypertrophic than in proliferative. On the contrary, miR-101a approximately falls by half in the hypertrophic period (Fig. 2A).

The ATDC5 cells, derived from the teratocarcinoma, are a common mouse chondrocyte cell line that could mimic the sequential process of endochondral ossification under the prolonged simulation culture of chondrogenic-induced medium containing ITS (insulin, transferrin, and sodium selenite).^34^^,^^40^^,^^41^ We further detect the expression of miR-101a, *Col10a1*, and *Cox-2* in ATDC5 cells with (ITS supplement, cultured for 21 days) or without (cultured for 0 day) hypertrophic-induced medium. The results indicated that the mRNA levels of *Col10a1* and *Cox-2* were both up-regulated 3-to-4-fold in hypertrophic ATDC5 cells compared with the proliferative ATDC5 cells. Meanwhile, the level of miR-101a was significantly decreased in hypertrophic chondrocytes (Fig. 2B). Collectively, these data suggest that miR-101a could be a potential negative regulator of *Col10a1* and *Cox-2*.

### miR-101a inhibits the expression of Col10a1 and Cox-2 both in MCT and ATDC5 cells

To confirm the effect of miR-101a on chondrocyte hypertrophy, three hypertrophic chondrocyte-related markers, *Col10a1*, *Cox-2*, and *Mmp-13*, were analyzed by quantitative PCR and western blotting after transfection with miR-101a mimic, miR-101a inhibitor, or corresponding negative controls. The results showed that the levels of *Cox-2* and *Col10a1* were significantly reduced after overexpression of miR-101a compared with the negative control in MCT and ATDC5 cells (Fig. 3A, B, D, E). However, there was no significant change in *Mmp-13* in MCT and ATDC5 cells (Fig. 3C–F). Meanwhile, silencing miR-101a in cell models of chondrocyte hypertrophy up-regulated *Col10a1* expression in MCT and ATDC5 cells. We also found that *Cox-2* and *Mmp-13* had high expression in ATDC5 cells, but no significant changes were found in *Cox-2* and *Mmp-13* in MCT cells (Fig. 3B–E). Western blotting results confirmed that miR-101a overexpression down-regulated the level of COL10A1, COX-2, and MMP13 (Fig. 3G and H). To support the reliability of these transfection experiments, we performed optimization of transfection concentrations for miR-101a mimic/inhibitor and a time-course analysis of miR-101a expression, as shown in Figure S1–S3. Together with the mRNA level, the results suggest that miR-101a could suppress the expression of *Col10a1*, *Cox-2*, and *Mmp-13* during chondrocyte hypertrophy.

### The effects of miR-101a on chondrogenic differentiation in ATDC5 cells

To further explore the influence of miR-101a on chondrocyte development, we performed three kinds of staining experiments on ATDC5 cells and quantitative reverse transcription PCR on several relevant marker genes during chondrocyte differentiation in MCT and ATDC5 cells.

As illustrated in Figure 4A, the intensity of Alcian blue staining was weaker in the miR-101a overexpression group than in the NC group, but no significant difference was observed in miR-101a-silencing ATDC5 cells, implying the partially positive role of miR-101a in chondrocyte differentiation and maturation. Nevertheless, no clear difference was observed in the intensity of Alizarin red staining and alkaline phosphatase staining between the experimental and control groups, indicating a limited role of miR-101a in chondrocyte proliferation at the early stage and matrix mineralization at the late stage of endochondral ossification (Fig. 4B and C). To further characterize the effect of miR-101a on chondrogenesis, we detected the expression of related transcription factors Runx2 and Sox9 by quantitative reverse transcription PCR and western blotting. However, there was no significant effect of miR-101a on Runx2 and Sox9 in hypertrophic MCT and ATDC5 cells induced by ITS for 21 days (Fig. 4D–F).

### Overexpression of miR-101a inhibits the proliferation ability of chondrocytes

We performed EdU assays to investigate the effect of miR-101a on the proliferation of chondrocytes. MCT and ATDC5 cells were seeded in 96-well plates at a density of 2.5 × 10^3^ cells per well. After 24 h, the cells were transfected with miR-101a mimics, miR-101a inhibitors, and negative controls separately. Transfection reagents were removed after 6 h, and cells were cultured for another 24 h in DMEM with 8% fetal bovine serum. The fluorescence results after merging EDU with DAPI indicated that forced miR-101a expression in MCT cells suppressed their proliferation, while inhibition of miR-101a expression facilitated the proliferation ability in chondrocytes (Fig. 5A), which is consistent with the results of Alcian blue staining. In addition, we also examined EdU cell proliferation in ATDC5 cells. After transient transfection with miR-101a inhibitor, it was able to partially promote the proliferation rate of ATDC5 cells, with no significant change after transfection with miR-101a mimic (Fig. 5B). Taken together, we found that miR-101a may have a role in inhibiting chondrocyte proliferation in *vitro*.

### Analysis and verification of miR-101a target sites

To investigate the specific mechanism of miR-101a between *Col10a1* and *Cox-2*, the dual-luciferase reporter assay was conducted. The potential binding site of miR101a with *Col10a1* and *Cox-2* predicted by TargetScan and the corresponding mutation sequences are shown in Figure 6A. Agarose gel electrophoresis was used to determine the size of the DNA fragments after PCR, and the fragment sizes were all located at 230 bp (Fig. 6B). After digesting and ligating the wild-type (WT) and mutation fragments to the pmirGLO vector, Sanger sequencing was used to confirm that the *Col10a1* and *Cox-2* vectors were constructed correctly and used for subsequent experiments (Fig. 6C and D). The reporter plasmid of pmirGLO-*Col10a1*-3′ UTR-WT and pmirGLO-*Col10a1*-3′ UTR-MUT was co-transfected with miR-101a mimic and mimic NC, respectively. The activity of luciferase of 293T cells was significantly reduced after being co-transfected with pmirGLO-*Cox-2*-3′ UTR-WT and miR101a mimic compared with the mutation vector. However, there was no significant difference in luciferase activity after transfection of pmirGLO-*Col10a1*-3′ UTR-WT and miR101a mimic (Fig. 6E). Together, the reporter assay showed that miR-101 could directly target the 3′ UTR region of *Cox-2*.

### Intra-articular injection of miR-101a attenuated OA progression

miRNA agomir and antagomir are chemically modified miRNA agonists and antagonists, respectively, which can be used to regulate target gene mRNA expression *in vivo*. Compared with common miRNA mimics and inhibitors, miRNA agomir and antagomir have higher stability and miRNA activity in animals, and are more likely to be enriched in target cells through cell membranes and tissue interstitial spaces. They can be administered systemically or locally, and their effects can last for several weeks.^37^^,^^38^

Figure 7A and B show the schematic and the timeline of the DMM surgery. The surgery was performed on the left knee joint of four-week-old C57BL/6 male mice, followed by intra-articular injections of 15 μL miR101a-agomir and control solution every 2 weeks after surgery. The mouse knee joint was collected at 8 weeks after the completion of the second injection for subsequent histological and molecular biological analysis. The hematoxylin and eosin staining of knee joint slides showed that the width of joint space became narrowed in the DMM group (Fig. 7C). The number of chondrocytes was significantly reduced in the DMM group (Fig. 7D). The safranin O/fast green staining and the statistical analysis of cartilage thickness illustrated that the cartilage damage and loss were most severe in the DMM group compared with the miRNA control group, and miR101-agomir supplement could partially alleviate cartilage damage (Fig. 7E and F).

### miR-101a inhibits Col10a1 and Cox-2 expression in the knee joints of DMM-induced OA mice

**Figure 8:**
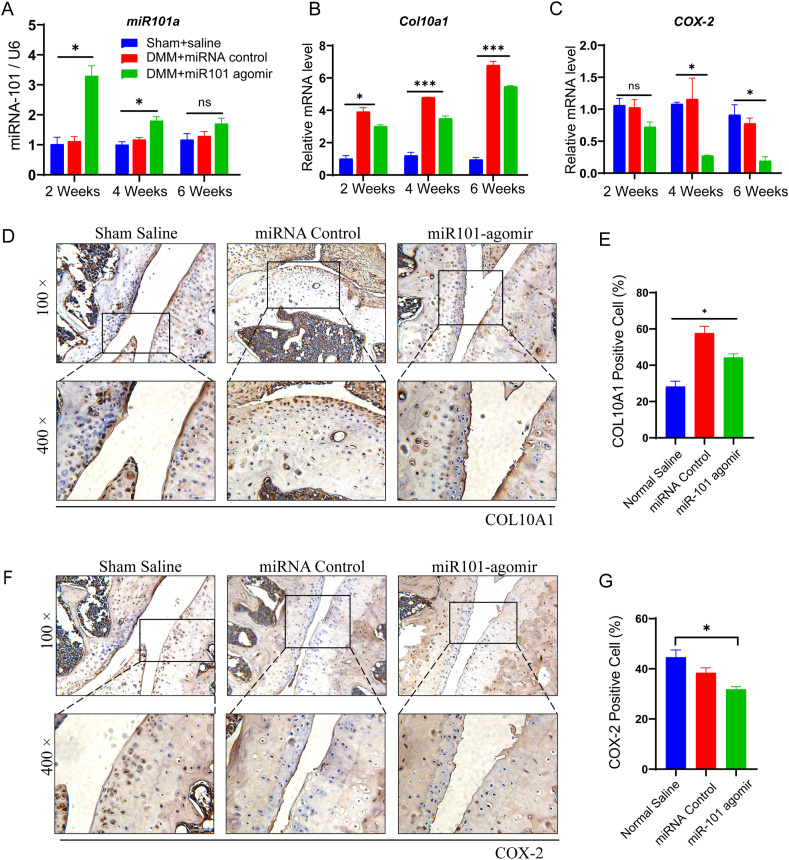
Col10a1, COX-2, and miRNA-101a expression after intra-articular injection of miRNA-101 agomir in DMM-induced OA mice. **(A**–**C)** The relative mRNA expression levels of *miRNA-101*, *Col10a1*, and *Cox-2* in mouse cartilage at 2, 4, and 6 weeks after intra-articular injection of miRNA-101 agomir (*n* = 2 in each group). *β-actin* and *U6* were used as endogenous controls. **(D)** The immunohistochemical staining of COL10A1 in the articular cartilage tissue of the knee joint. **(E)** Quantification of the percentage of COL10A1-positive cells in the three groups as determined by the staining results. **(F)** The immunohistochemical staining of COX-2 in the articular cartilage tissue of the knee joint (*n* = 4 in each group). Magnification, 100 × or 400 × . **(G)** Quantification of the percentage of COX-2 positive cells in the three groups as determined by the staining results. ∗*P* < 0.05, ∗∗*P* < 0.01, and ∗∗∗*P* < 0.001.

According to the surgical timeline settings shown in Figure 7A, the knee joint tissues were harvested at 2 weeks, 4 weeks, and 6 weeks after the second injection, respectively. Quantitative PCR analysis and immunohistochemical staining were conducted to assess the expression level of *Col10a1* and *Cox-2*. The mRNA level of miR101a was increased in the first 4 weeks after injection (Fig. 8A). The mRNA expression of *Col10a1* in the DMM group was extremely evaluated and the addition of miR101-agomir partially decreased the level of *Col10a1* (Fig. 8B). The mRNA of *Cox-2* did not show significant differences between the DMM and sham groups, however, miR101-agomir could inhibit the expression of *Cox-2* significantly (Fig. 8C). Meanwhile, the immunohistochemical staining of COL10A1 showed an increased protein level in the DMM group compared with the sham group, and the proportion of positive cells in the superficial layer of articular cartilage in the group with intra-articular injection of miR101-agomir was decreased than that in the miRNA-agomir control group (Fig. 8D and E). However, compared with the sham group, there was a decrease in the protein level of COX-2 in the DMM group, and the injection of agomir had no significance between the miR-101a and negative control groups (Fig. 8F and G). The immunohistochemical staining and the proportion of the positive cells in articular cartilage tissues of OA mice suggest that miR-101a could inhibit the degradation of type X collagen.

## Discussion

The process of cartilage development and homeostasis is tightly regulated by a coordinated network of transcription factors, signaling molecules, and epigenetic regulators. Among these, *Cox-2* and *Col10a1* play essential roles during chondrocyte hypertrophy.^24^ While the epigenetic regulation of hypertrophy-related genes by miRNAs has been increasingly recognized, specific interactions remain underexplored. For instance, although *miR-101a* has previously been shown to regulate extracellular matrix-degrading genes and *Sox9* in cartilage,^32^ its role in modulating hypertrophic differentiation has not been well defined. In this study, we explored how *miR-101a* influenced hypertrophic chondrocyte marker expression. We observed that *miR-101a* expression was down-regulated during the hypertrophic stage in MCT and ATDC5 cells, while *Cox-2* and *Col10a1* were significantly up-regulated. Overexpression of *miR-101a* suppressed both *Cox-2* and *Col10a1*, along with *Mmp-13*, an extracellular matrix-degrading enzyme involved in OA progression.^42^ These results suggest that *miR-101a* acts as a negative regulator of chondrocyte hypertrophy.

Interestingly, *miR-101a* did not significantly affect *Runx2* or *Sox9* expression in our system, suggesting that it may act upstream of or parallel to these master transcription factors. Given that each miRNA can target hundreds of mRNAs,^43^ the context-specific regulation of *miR-101a* is not surprising. Previous studies in OA models showed that *miR-101a* suppressed chondrocyte proliferation,^32^^,^^44^ consistent with our incorporation results of Alcian blue and EdU stainings, which indicate reduced proliferation upon *miR-101a* overexpression. These findings support a model in which *miR-101a* may exert distinct regulatory effects at different stages of chondrocyte development. To determine direct molecular interactions, we conducted luciferase reporter assays and confirmed *Cox-2* as a direct target of *miR-101a*. Previous work in other cell types has shown that *miR-101a* represses *Cox-2* expression, including in mammary gland epithelial cells, colon cancer, and esophageal squamous-cell carcinoma cell lines.^45^^,^^46^ Our findings extend this regulatory relationship to chondrocytes and further link it to hypertrophy.

Mechanistically, our data suggest that *miR-101a* may delay chondrocyte hypertrophy by targeting *Cox-2*, which plays a key role in prostaglandin E2 (PGE2) synthesis. PGE2 signaling has been implicated in promoting hypertrophic differentiation via Runx2 activation. Notably, *Cox-2* has been shown to act as a co-activator of Runx2, enhancing its transcriptional activity and thereby up-regulating *Col10a1* expression.^47^^,^^48^ Moreover, *Cox-2* activity is essential for bone morphogenetic protein 2 (BMP-2)-mediated hypertrophy, further supporting its role in this pathway. Therefore, *miR-101a*-mediated suppression of *Cox-2* could reduce PGE2 levels, inhibit Runx2 activity, and subsequently down-regulate *Col10a1*. This highlights a potential *miR-101a–Cox-2–PGE2–Runx2–Col10a1* axis that regulates hypertrophic differentiation. Future studies using pathway inhibitors or genetic manipulation of downstream targets will be important to confirm this regulatory network. Although our findings support the therapeutic potential of *miR-101a* in OA, the clinical translation of miRNA-based therapies remains challenging. A recent study showed that intra-articular delivery of miR-15a promoted chondrocyte apoptosis in OA mice by targeting B-cell lymphoma 2 (*BCL-2*), illustrating both the potential and complexity of miRNA-based interventions.^49^ In our model, intra-articular injection of *miR-101a* mimics resulted in reduced *Col10a1* expression and cartilage degradation, suggesting a protective role in OA.

We acknowledge several limitations in our study. First, the lack of a positive control drug precludes a direct assessment of *miR-101a*′s therapeutic efficacy. Second, although micro–computed tomography (micro-CT) offers a non-destructive method to quantify cartilage volume, subchondral bone remodeling, and osteophyte formation in OA models,^50^ we were unable to perform this analysis because the original animal studies had already concluded and the samples had subsequently degraded. To address these limitations, future studies will include both micro-CT imaging and a positive drug control to rigorously validate the therapeutic potential of *miR-101a*. In parallel, we are investigating the mechanistic role of *miR-101a* in BMP-2-induced C2C12 cells, a well-established model of osteochondral differentiation. These complementary studies will clarify whether *miR-101a* exerts broader regulatory functions across distinct stages and models of cartilage development and degeneration (supplemental data and data not shown).

## Conclusions

In summary, our findings identify *miR-101a* as a novel post-transcriptional regulator of chondrocyte hypertrophic differentiation through direct targeting of *Cox-2*, leading to downstream suppression of *Col10a1* expression. This *miR-101a–Cox-2–Col10a1* axis may play a critical role in delaying hypertrophy and attenuating OA cartilage degeneration. While further studies are needed to delineate the precise downstream signaling mechanisms and validate therapeutic efficacy in more comprehensive models, our study provides new insight into the epigenetic regulation of OA progression and highlights *miR-101a* as a promising target for future therapeutic development.

## CRediT authorship contribution statement

**Rui Mi:** Writing – original draft, Conceptualization, Data curation. **Jinnan Chen:** Methodology, Data curation, Writing – original draft, Formal analysis, Conceptualization. **Tianxiang Zhu:** Formal analysis, Methodology. **Huiqin Bian:** Data curation, Methodology. **Rong Wei:** Writing – review & editing. **Rushuang Deng:** Writing – review & editing. **Tiaotiao Han:** Methodology. **Qian Wang:** Data curation. **Yaojuan Lu:** Writing – review & editing. **Longwei Qiao:** Writing – review & editing. **Yuting Liang:** Writing – original draft. **Qiping Zheng:** Writing – review & editing, Investigation, Conceptualization, Supervision, Funding acquisition.

## Data availability

All data generated or analyzed during this study are included in this published article.

## Ethics declaration

All experiments and methods were performed according to relevant guidelines and regulations. All animal procedures were approved by the Institutional Animal Care and Use Committee of Jiangsu University.

## Funding

This work was supported by the 10.13039/501100013058Jiangsu Provincial Key Research and Development Program (No. BE2020679 to Q.Z.), the Innovation Team (leader) of Jiangsu Province, China (2017, to Q.Z.), Science Foundation of Jiangsu Province (China) (No. BK20240371 to Y.L.), and the National Science Foundation of China (No. 81901632 to Y.L.; 82001576 to L.Q.).

## Conflict of interests

The authors declared no conflict of interests.
