# Supplementary material for: Role of miR-101a in targeting Cox-2 to attenuate chondrocyte hypertrophic differentiation and osteoarthritis progression
Source: Genes Dis. 2025 Sep 1;13(3):101839. doi: 10.1016/j.gendis.2025.101839 (PMC12886533; doi:10.1016/j.gendis.2025.101839)
Supplement: Multimedia component 1 [file mmc1.docx]

**Below are the supplementary figures**

**Supplemental Figure 1: Optimization of miR-101a-3p transfection in MCT and ATDC5 cells.**


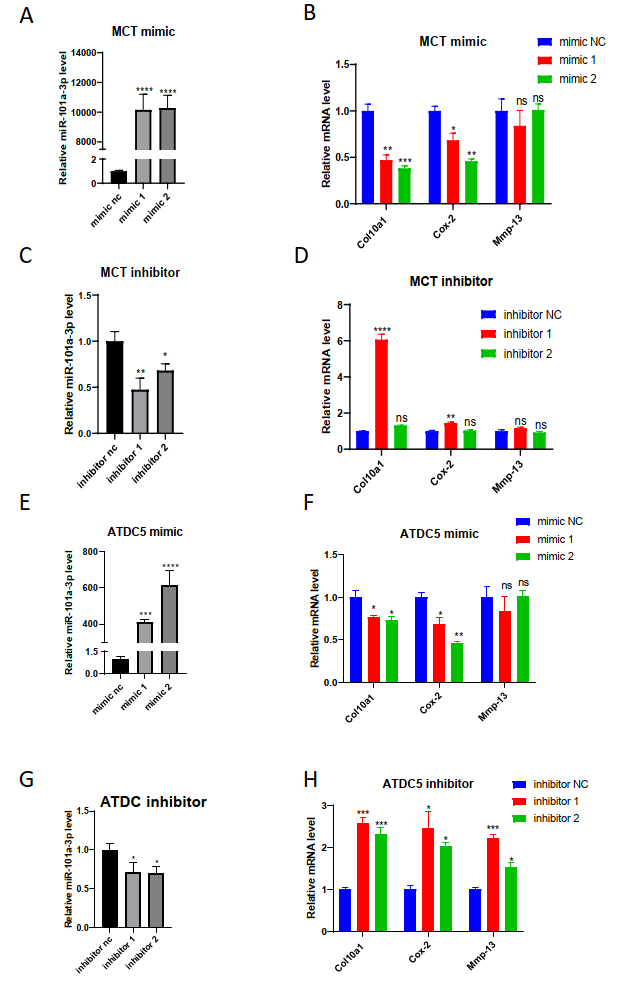


**Supplemental Figure 2: Time-course of miR-101a-3p and downstream gene expression under hypertrophic induction**


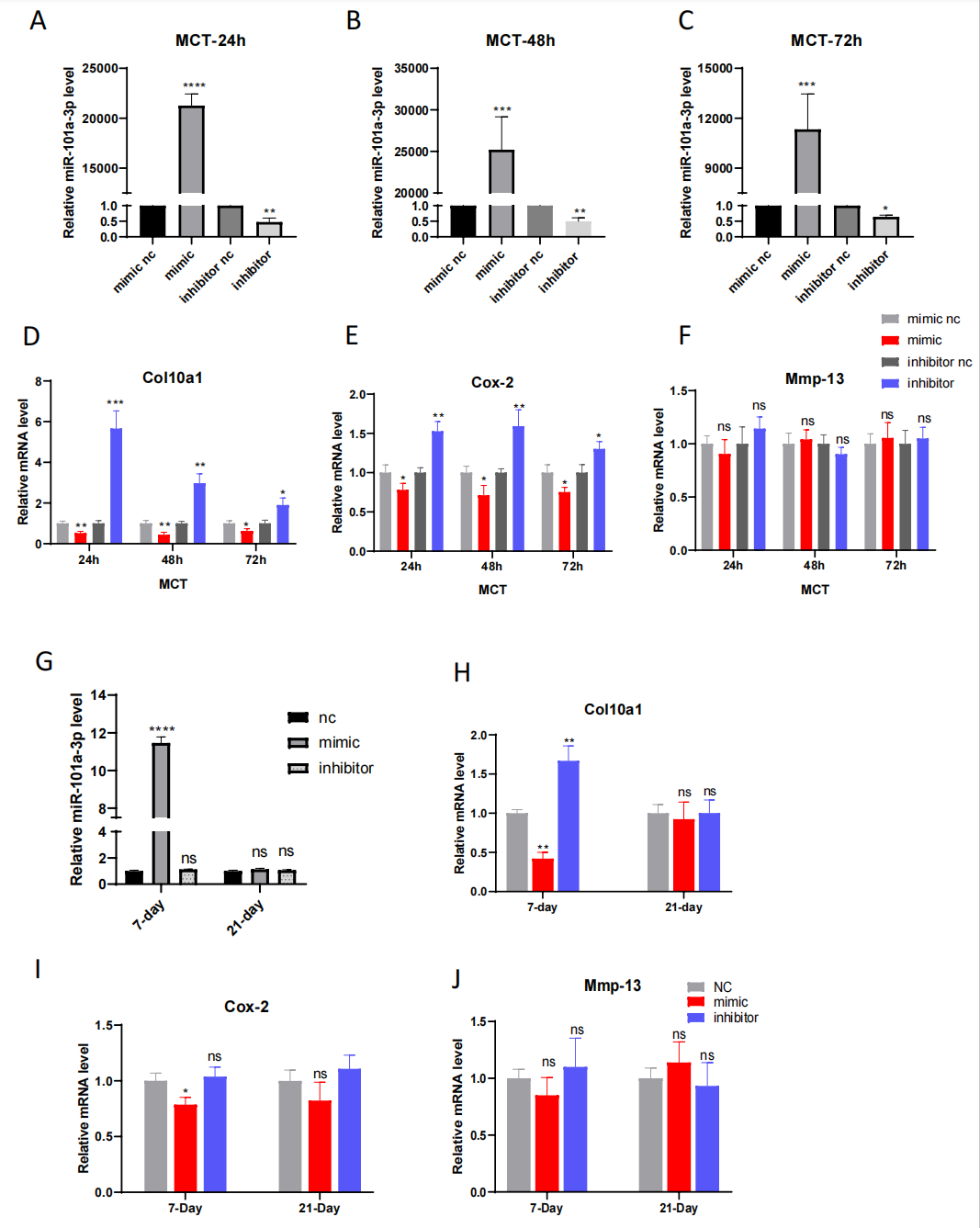


**Supplemental Figure 3 Protein expression of COX-2 and COL10A1 after miR-101a-3p modulation**

**
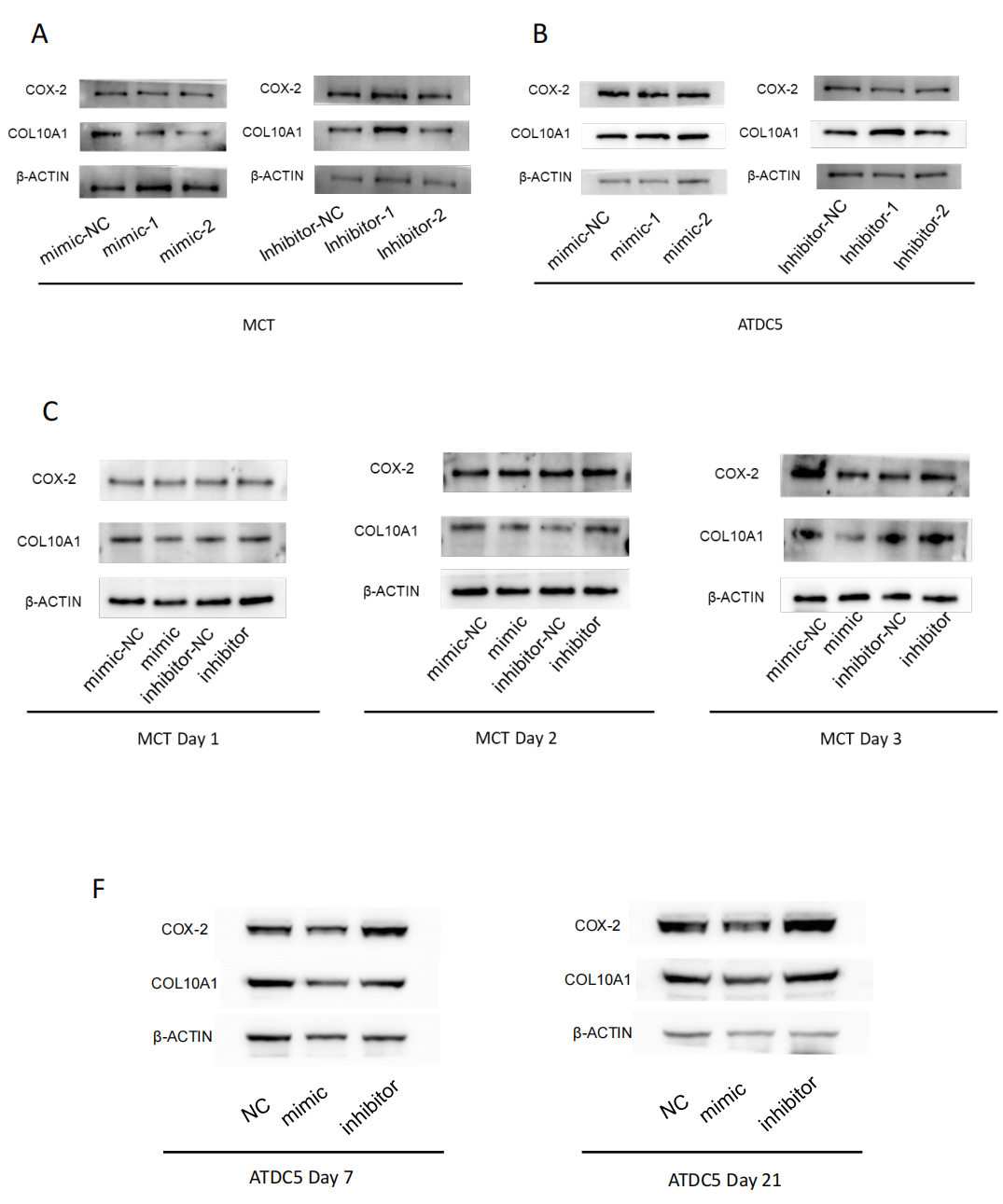
**

**Supplementary Figure 1. Optimization of miR-101a-3p transfection efficiency and downstream gene regulation in MCT and ATDC5 cells.**

MCT and ATDC5 chondrocyte cell lines were transfected with different concentrations of miR-101a-3p mimic (40 nM and 80 nM) or inhibitor (80 nM and 120 nM). Cells transfected with negative control oligonucleotides (NC) served as controls.

(A, C) qRT-PCR analysis of miR-101a-3p expression following transfection with mimics (A) or inhibitors (C) in MCT cells. (B, D) mRNA expression of *Col10a1*, *Cox-2*, and *Mmp13* in MCT cells after treatment with mimics (B) or inhibitors (D). (E, G) qRT-PCR analysis of miR-101a-3p expression following transfection with mimics (E) or inhibitors (G) in ATDC5 cells. (F, H) mRNA expression of *Col10a1*, *Cox-2*, and *Mmp13* in ATDC5 cells after treatment with mimics (F) or inhibitors (H). All gene expression levels were normalized to U6 (for miRNA) or Actin (for mRNA). Data are shown as mean ± SEM (n = 3). **P* < 0.05*; **P* < 0.01*; ***P* < 0.001; *****P* < 0.0001; ns, not significant (Student’s t-test or one-way ANOVA with post hoc test as appropriate).

**Supplementary Figure 2. Time-course analysis of miR-101a-3p expression and downstream gene regulation in MCT and ATDC5 cells under hypertrophic induction.**

MCT cells were transfected with miR-101a-3p mimic (80 nM) or inhibitor (80 nM), and cultured under hypertrophic conditions for 24, 48, or 72 hours, while ATDC5 cells were treated similarly (mimic: 80 nM, inhibitor: 120 nM) and collected at day 7 and day 21 of ITS-induced hypertrophic differentiation.

(A–C) qRT-PCR analysis of miR-101a-3p expression at each time point. (D–F) mRNA levels of *Col10a1* (D), *Cox-2* (E), and *Mmp13* (F) in MCT cells at each time point. (G) qRT-PCR analysis of miR-101a-3p expression in ATDC5 cells on day 7 and day 21 of ITS-induced hypertrophic differentiation after transfection with mimic (80 nM) or inhibitor (120 nM).; (H–J) mRNA levels of *Col10a1* (H), *Cox-2* (I), and *Mmp13* (J). Data are shown as mean ± SEM (n = 3), normalized to U6 (miRNA) or Actin (mRNA). **P* < 0.05; ***P* < 0.01; ****P* < 0.001; *****P* < 0.0001; ns, not significant.

**Supplementary Figure 3. Protein expression of COX-2 and COL10A1 in MCT and ATDC5 cells after miR-101a-3p mimic or inhibitor transfection.**

(A, B) MCT (A) and ATDC5 (B) cells were transfected with two concentrations of miR-101a-3p mimics or inhibitors. Western blot was performed to detect COX-2 and COL10A1 protein levels; β-ACTIN served as a loading control. (C) MCT cells were transfected with mimic (80 nM) or inhibitor (80 nM) and cultured under hypertrophic conditions for 24, 48, and 72 h. Protein levels of COX-2 and COL10A1 were evaluated at each time point. (F) ATDC5 cells were transfected with mimic (80 nM) or inhibitor (120 nM) and harvested on day 7 and day 21 of ITS-induced hypertrophic differentiation. COX-2 and COL10A1 expression was assessed by Western blot.
